# Dual Antiglutamate Therapy in Super-Refractory Status Epilepticus After Cardiac Arrest (SUPER-CAT Study)

**DOI:** 10.1212/WNL.0000000000218565

**Published:** 2026-10-07

**Authors:** Simone Beretta, Matteo Pozzi, Simone Tagliabue, Elisa Bianchi, Emanuela Rossi, Francesco Pasini, Angela Giglio, Giulia Sofia Cereda, Carlo Morotti Colleoni, Giulia Pederzoli, Giada Giovannini, Niccolò Orlandi, Stefano Meletti, Alessandra Gaiani, Stefania Filipponi, Alberto Cucino, Marco Normanno, Ilaria Mariani, Margherita Scanziani, Marco Giani, Susanna Diamanti, Anna Coppo, Jacopo C. DiFrancesco, Andrea Stabile, Lorenzo Tinti, Igor Florio, Chiara Sist, Marta Corona, Beatrice Pinna, Francesco Orlandini, Giovanni Cossu, Monica Ferlisi, Marilena Casartelli-Liviero, Francesco Misirocchi, Irene Florindo, Lucia Zinno, Yerma Bartolini, Marco Longoni, Emilio Russo, Antonello Grippo, Carlo Ferrarese, Giuseppe Foti, Leonello Avalli

**Affiliations:** 1 Epilepsy Center, Department of Neurology, Fondazione IRCCS San Gerardo dei Tintori, Monza, Italy;; 2 Department of Medicine and Surgery, University of Milano-Bicocca, Italy;; 3 Department of Emergency and Intensive Care, Fondazione IRCCS San Gerardo dei Tintori, Monza, Italy;; 4 Istituto di Ricerche Farmacologiche Mario Negri IRCCS, Milano, Italy;; 5 Epilepsy Unit, Fondazione IRCCS Istituto Neurologico Carlo Besta, Milano, Italy;; 6 PhD Program in Neuroscience, University of Milano-Bicocca, Milano, Italy;; 7 Neurophysiology Unit and Epilepsy Centre, Ospedale Civile di Baggiovara, Azienda Ospedaliero-Universitaria di Modena, Italy;; 8 Department of Biomedical, Metabolic and Neural Sciences, University of Modena and Reggio Emilia, Italy;; 9 Department of Neurology and Intensive Care, Santa Chiara Hospital, Azienda sanitaria universitaria integrata del Trentino (Asuit), Trento, Italy;; 10 Epilepsy Unit, IRCCS Mondino Foundation, Pavia, Italy;; 11 S.C. Neurologia, Ospedale Centrale di Bolzano, Italy;; 12 S.C. Neurologia, Ospedale Brotzu Cagliari, Italy;; 13 Neurology Unit, Department of Neurosciences, Azienda Ospedaliera Universitaria Integrata, Verona, Italy;; 14 Division of Anesthesia and Intensive Care, Azienda Ospedaliera Universitaria Integrata, Verona, Italy;; 15 Unit of Neurology, University Hospital of Parma, Italy;; 16 S.C. Neurologia, Ospedale M. Bufalini Cesena, Italy;; 17 CRUISE Research Center, Dipartimento di Scienze della Salute, Università Magna Graecia di Catanzaro, Italy; and; 18 S.C. Neurofisiologia, Azienda Ospedaliero-Universitaria Careggi, Firenze, Italy.

## Abstract

**Background and Objectives:**

The SUPER-refractory status epilepticus (SE) after Cardiac Arrest Treatment (SUPER-CAT) study evaluated the efficacy and safety of dual antiglutamate therapy (combined AMPA and NMDA receptor blockade) in patients with super-refractory SE (SRSE) after cardiac arrest and without unfavorable prognostic indicators.

**Methods:**

Multicenter cohort study including patients with postanoxic SRSE meeting the following criteria: bilaterally preserved N20 cortical responses, intact pupillary reflexes, and absence of generalized periodic discharges. Patients treated with dual antiglutamate therapy (DUAL; ketamine 1.5–7.5 mg/kg/h for 3 days plus perampanel 6–12 mg/d for 5 days with tapering) were compared with patients receiving other combinations of antiseizure and anesthetic agents (OTHERS). The primary outcome was resolution of SE. Secondary outcomes included awakening to follow commands and good neurologic outcome at 6 months (modified Rankin Scale 0–3) in patients with fewer than 2 unfavorable multimodal prognostic indicators and 6-month mortality for the entire cohort. Safety analysis included in-hospital mortality and potentially treatment-related adverse events. Patients treated with single antiglutamate agents were described separately.

**Results:**

Between May 2012 and May 2025, 95 of 1,352 consecutive post–cardiac arrest intensive care unit patients met inclusion criteria (mean age 66, interquartile range 55–73; 41% female; DUAL = 43; OTHERS = 41; single antiglutamate = 11). Compared with OTHERS, DUAL was associated with a higher rate of SE resolution (84% vs 49%; OR 5.4; 95% CI 1.9–14.9; *p* = 0.001), a greater likelihood of awakening before discharge (56% vs 32%; OR 2.7; 95% CI 1.0–7.3; *p* = 0.053) and lower 6-month mortality (52% vs 74%; OR 0.37; 95% CI 0.15–0.97; *p* = 0.043). A trend toward better 6-month neurologic outcome was observed in the DUAL group (39% vs 25%; OR 1.89; 95% CI 0.64–5.56; *p* = 0.245). Reversible cholestatic liver injury occurred more frequently in DUAL group, compared with OTHERS (77% vs 27%; OR 9.00; 95% CI 3.34–24.19; *p* < 0.001).

**Discussion:**

In patients with postanoxic SRSE without unfavorable prognostic indicators, dual antiglutamate therapy was highly effective in resolving SE and was associated with improved short-term clinical outcomes.

**Trial Registration Information:**

ClinicalTrials.gov NCT05756621.

## Introduction

Status epilepticus (SE) is a frequent and challenging complication after cardiac arrest, affecting up to 20%–30% of comatose survivors and strongly influencing neurologic outcomes.^1^ Postanoxic SE is often refractory to standard antiseizure therapy and may frequently progress to super-refractory SE (SRSE), defined as seizure activity persisting or recurring >24 hours after initiation of a combination of antiseizure medications (ASMs) and anesthetics.^2^ The optimal therapeutic regimen, intensity, and duration for postanoxic SRSE remains controversial, particularly given concerns about treatment futility.^3-7^ Excitotoxicity driven by excessive glutamate release plays a central role in postanoxic neuronal injury, with overactivation of N-methyl-D-aspartate (NMDA) and α-amino-3-hydroxy-5-methyl-4-isoxazolepropionic acid (AMPA)-mediated receptors promoting ongoing seizure activity.^8,9^ This provides a rationale for glutamate-targeted therapies.

Current European Resuscitation Council (ERC) guidelines^10^ support multimodal prognostication using neurologic examination, EEG, median nerve somatosensory evoked potentials (SSEPs), neuron-specific enolase (NSE), and neuroimaging but offer limited guidance on second-line antiseizure treatment and do not specifically address SRSE. Consequently, the role of aggressive antiseizure therapy in this context remains debated and is generally guided by individualized clinical judgment. Although the Treatment of ELectroencephalographic STatus Epilepticus After Cardiopulmonary Resuscitation (TELSTAR) trial showed futility of aggressive treatment of generalized periodic discharges (GPDs),^11^ its findings may not apply to patients meeting Salzburg criteria for definite SE.^12^ Previous observational studies from independent groups indicated that active antiseizure treatment may yield favorable outcomes in selected patients.^13-16^

Given their complementary mechanisms of action, ketamine and perampanel offer a targeted strategy against NMDA-mediated and AMPA-mediated excitotoxicity.^17^ The SUPER-refractory SE after Cardiac Arrest Treatment (SUPER-CAT) multicenter cohort study evaluated the efficacy and safety of combined antiglutamate therapy for postanoxic SRSE in patients without unfavorable prognostic indicators.

## Methods

### Study Design and Setting

The SUPER-CAT study was a multicenter, observational cohort study, conducted across 8 intensive care units (ICUs) and epilepsy centers in Italy, including retrospective data from May 2012 to September 2022 and prospective data from October 2022 to May 2025. Patients were treated according to standard clinical practice; no treatments were assigned for research purposes. Clinical data and outcomes were collected from existing records. Data were captured using electronic case report forms (eCRFs) on the Research Electronic Data Capture Cloud platform.^18^

### Standard Protocol Approvals, Registrations, and Patient Consents

The study protocol, covering both the retrospective and prospective phases, was approved by the Ethics Committee at each participating center. The study was registered at ClinicalTrials.gov (NCT05756621). A single informed consent form was used. For retrospective cases, informed consent was obtained when feasible from living, contactable patients. For prospective patients, informed consent was obtained prospectively whenever possible; if patients died or never regained decision-making capacity, informed consent was waived. Written informed consent was obtained from patients or their legally authorized representatives. In emergency cases, deferred consent was applied in accordance with national regulations. For deceased or noncontactable patients, consent waiver was granted in accordance with Article 21 of Legislative Decree 101/2018 and Article 110 of the Italian Data Protection Code.

### Study Participants

We included consecutive adult patients who experienced cardiac arrest and were admitted to the ICU between May 2012 and May 2025; detailed enrolment periods for each participating center are presented in eTable 1. Eligible patients were comatose after cardiac arrest and met the following criteria: They underwent targeted temperature management during the first 24 hours, had continuous EEG monitoring initiated within 36 hours of the event, fulfilled Salzburg criteria^12^ for definite SRSE, and had preserved bilateral pupillary reflexes as well as bilateral cortical N20 responses on median nerve SSEPs. Patients were excluded if GPDs were present on EEG at the time of initial electrographic diagnosis or the first recurrence of SE. GPDs were defined according to the American Clinical Neurophysiology Society (ACNS) critical care EEG terminology^19^ and, for the purpose of exclusion, were specifically <2.5 Hz and nonevolving. Additional exclusion criteria were resolution of SE after the first cycle of anesthetics and ASMs, age younger than 18 years, or pregnancy.

### Treatments

Patients in the DUAL group received dual antiglutamate therapy consisting of the simultaneous initiation, on the same day, of continuous intravenous ketamine and oral perampanel. This treatment protocol, termed SUPER-CAT, was initially developed at the coordinating center based on preliminary clinical experience^20^ and was subsequently adopted by the other participating centers. Ketamine was administered intravenously for 3 days, with dosing determined according to local clinical practice (induction dose: 1.5–3 mg/kg, followed by continuous infusion at 1.5–7.5 mg/kg/h). Ketamine dosing was titrated according to continuous EEG monitoring to achieve a continuous alpha-beta background without electrographic seizure activity. Intravenous midazolam (1–15 mg/h) was coadministered as needed. Perampanel was administered for 5 days using a nasogastric tube as crushed tablets (dose range, 6–12 mg). Perampanel was subsequently tapered by 2 mg every 2 days and discontinued if no electrographic or clinical seizure recurrence was observed.

Patients in the OTHERS group received conventional ASMs and anesthetic therapies according to standard clinical practice, of any type and dose, except ketamine and perampanel. Additional cohorts receiving single-agent antiglutamatergic therapy, ketamine alone (SINGLE-K) or perampanel alone (SINGLE-P), were analyzed separately.

### Study Outcomes

The primary outcome was resolution of SE without recurrence and without the need for additional ASMs or anesthetics, assessed throughout the entire ICU stay. The duration of continuous EEG monitoring after SE resolution was not predefined and varied across centers. All available EEG tracings obtained during the ICU stay were used to adjudicate resolution vs recurrence.

Secondary outcomes included the cumulative probability of SE resolution; early and long-term good neurologic outcomes, defined as awakening to the ability to follow commands before hospital discharge and a modified Rankin Scale (mRS) score^21^ of 0–3 at 6 months, both assessed in patients with fewer than 2 unfavorable prognostic indicators according to ERC guidelines^10^; and 6-month mortality for the entire cohort. Recovery of consciousness up to command following was assessed through review of medical records. The mRS was assessed by either phone interview or in-person visit for both retrospective and prospective patients. For retrospective patients, the assessment referred to neurologic disability at 6 months after the event. Safety outcomes included mortality in the ICU, elevation of gamma-glutamyl transferase (GGT) greater than 3 times the upper normal limit (UNL), creatine kinase (CK) greater than 10 times the UNL, third-degree atrioventricular block, recurrence of cardiac arrest, and any potentially treatment-related adverse events.

### Sample Size Estimate

Preliminary data suggested that SRSE resolution occurred in approximately 20% of patients treated with conventional therapies and in approximately 60% of those receiving dual antiglutamate therapy. Based on these estimates, a sample size of 80 patients (40 per group) was calculated to provide 80% power to detect a conservative 30% absolute difference in SRSE resolution, with a 2-tailed α of 0.05. The sample size estimate served to assess statistical power for the overall cohort and guide the prospective enrollment.

### Statistical Analysis

Descriptive statistics were used to summarize baseline characteristics, with frequencies and percentages for categorical variables and medians (interquartile range, IQR) for continuous variables. Between-group comparisons (DUAL vs OTHERS) used χ^2^ or Fisher exact tests for categorical variables and *t* tests or Wilcoxon-Mann-Whitney tests for continuous variables. The primary efficacy, secondary, and safety outcomes were compared between treatment groups using χ^2^ tests. Treatment-outcome associations were evaluated using logistic regression models with treatment group as the only independent variable, given the limited sample size. The results are reported as odds ratios (ORs) with 95% CIs. The cumulative probability of SE resolution and awakening was analyzed using cumulative incidence functions accounting for death as competing event, with Gray test for between-group comparisons. Six-month survival was analyzed using Kaplan-Meier curves and compared between groups using the log-rank test. Descriptive analyses for the SINGLE-K and SINGLE-P groups were reported for each outcome. Statistical significance was set at *p* < 0.05 (2-tailed). Missing data were not imputed, including data missing due to loss to follow-up. Analyses were performed using SAS software (version 9.4, SAS Institute, Cary, NC).

### Data Availability

Qualified investigators may request access to anonymized individual patient-level data and relevant trial documentation, including raw and analysis-ready data sets, the study protocol, annotated eCRFs, the statistical analysis plan, and dataset specifications. Requests will be evaluated by the SUPER-CAT Steering Committee. Upon approval, access to the data will be granted after execution of a formal data sharing agreement, in accordance with applicable ethical and regulatory requirements.

## Results

### Study Population and Neuroprognostic Indicators

Among 1,352 patients admitted to the ICU after cardiac arrest during the study period, 95 (7.0%) met inclusion criteria. Of these, 84 patients were included in the 2 primary comparison groups (DUAL and OTHERS), while 11 patients treated with single antiglutamate agents were analyzed separately (eFigures 1 and 2). Baseline demographic and clinical characteristics were generally balanced between DUAL and OTHERS (Table 1). The median age was 64 years (IQR 53–71) in the DUAL group and 69 years (IQR 60–73) in the OTHERS group. Men accounted for 48.8% and 65.8% of patients, respectively. Out-of-hospital cardiac arrest occurred in 62.8% vs 75.6% and shockable rhythms in 51.2% vs 63.4%. The median low-flow times (21 vs 22 minutes), clinical myoclonus (27.9% vs 21.9%), and NSE level (45 vs 31 μg/L) were comparable, as well as the prevalence of preserved corneal reflexes (23% vs 23%) and diffuse anoxic injury on imaging (13% vs 9%).

**Table 1:** Clinical Characteristics and Neuroprognostic Indicators

	DUAL (n = 43)	OTHERS (n = 41)	*p* Value
Age, y, median (IQR)	64 (53–71)	69 (60–73)	0.154
Sex, male, n (%)	21 (48.8)	27 (65.8)	0.129
OHCA, n (%)	27 (62.8)	31 (75.6)	0.242
Shockable rhythm, n (%)	22 (51.2)	26 (63.4)	0.278
No-flow time, min, median (IQR)	0 (0)	0 (0–1)	1.000
Low-flow time, min, median (IQR)	21 (10–35)	22 (12–30)	0.590
Corneal reflex present, n (%)	23 (53.4)	23 (56.1)	1.000
NSE (highest value 24–72 h), μg/L, median (IQR)	45 (27–65)	31 (20–63)	0.830
Diffuse anoxic injury at CT or MRI, n (%)	13 (30.2)	9 (28.1)	0.804
Clinical myoclonus, n (%)	12 (27.9)	9 (21.9)	0.618

Abbreviations: IQR = interquartile range; OHCA = out of hospital cardiac arrest; NSE = neuron-specific enolase.

### Therapeutic Exposure

Treatment characteristics are summarized in Table 2. All patients in the DUAL group received continuous intravenous ketamine for 3 days (median dose 5 mg/kg/h, IQR 4–6), titrated to achieve the target ketamine EEG pattern (Figure 1B). Intravenous midazolam was coadministered in 30 patients (69.8%; median dose 2 mg/h, IQR 0–5). Oral perampanel (median dose 12 mg, IQR 8–12) was administered concomitantly for 5 days and subsequently tapered. Patients in both groups received valproate (88% vs 85%), levetiracetam (67% vs 70%), and propofol (97% vs 98%) in comparable proportions. Other ASMs used during the ICU stay are listed in eTable 2. Patients in the DUAL group were less likely to receive additional ASMs (39% vs 53%), thiopental (14% vs 27%), and midazolam (63% vs 83%) than those in the OTHERS group. The median number of ASMs was higher in the DUAL group (3 [IQR 2–4] vs 2 [2–3]), as well as the median number of anesthetic cycles (3 [2–3] vs 2 [2–3]). In 75 of 84 patients (89.3%), anesthetic agents other than ketamine, including those administered before the super-refractory phase of SE, were titrated to achieve EEG burst suppression. Duration of ICU stay was longer in the DUAL group (33 vs 15 days).

**Table 2:** ASMs and Anesthetics Received During the ICU Stay

	DUAL (n = 43)	OTHERS (n = 41)
No. of ASMs, median (IQR)	3 (2–4)	2 (2–3)
No. of anesthetic cycles, median (IQR)	3 (2–3)	2 (2–3)
Perampanel dose, mg, median (IQR)	12 (8–12)	0 (0)
Ketamine (EEG-targeted dose), mg/kg/h, median (IQR)	5 (4–6)	0 (0)
Valproate, n (%)	38 (88)	35 (85)
Levetiracetam, n (%)	29 (67)	29 (70)
Other ASMs, n (%)^a^	17 (39)	22 (53)
Propofol, n (%)	42 (97)	40 (98)
Midazolam, n (%)	27 (63)	34 (83)
Thiopental, n (%)	6 (14)	11 (27)
Duration of ICU stay, d, median (IQR)	33 (26–51)	15 (9–24)

Abbreviations: ASM = antiseizure medication; ICU = intensive care unit; IQR = interquartile range.

a See eTable 2 for details.

**Figure 1:**
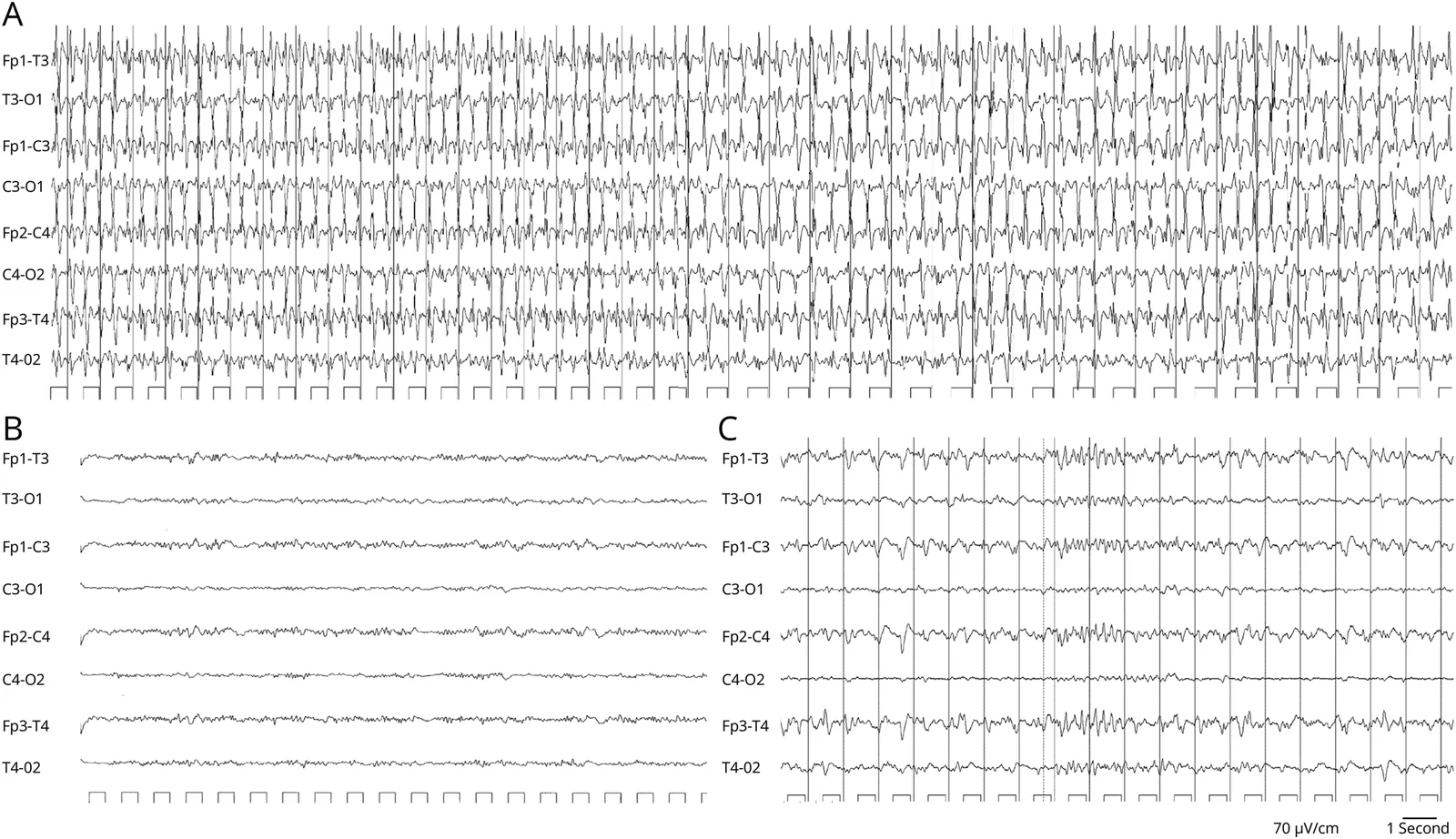
EEG Course in a Representative Postanoxic Patient With Super-Refractory Status Epilepticus EEG epochs are shown before (A), during (B), and 5 days after (C) dual antiglutamate treatment.

### Primary Outcome

Resolution of SRSE during the ICU stay occurred in 36 of 43 patients (83.7%) in the DUAL group and 20 of 41 patients (48.8%) in the OTHERS group (OR 5.40; 95% CI 1.95–14.90; *p* = 0.001; Table 3). Representative EEG course in a postanoxic patient treated with DUAL therapy is illustrated in Figure 1. The cumulative probability of SE resolution over 30 days, with Gray test indicating a significantly higher probability in the DUAL group (*p* = 0.006), is shown in Figure 2.

**Table 3:** Primary and Secondary Outcomes

	DUAL (n = 43)	OTHERS (n = 41)	OR (95% CI)	*p* Value
Primary outcome				
Resolution of status epilepticus during the ICU stay, n/N (%)	36/43 (83.7)	20/41 (48.8)	5.40 (1.95–14.90)	0.001
Secondary outcomes				
Early good neurologic outcome^a^ (awakening to follow commands during the ICU stay), n/N (%)	18/32 (56.2)	11/34 (32.3)	2.68 (0.99–7.32)	0.053
Long-term good neurologic outcome^a^ (mRS 0–3 at 6 mo), n/N (%)	12/31 (38.7)	8/32 (25.0)	1.89 (0.64–5.56)	0.245
Mortality at 6 mo, n/N (%)	22/42 (52.4)	29/39 (74.4)	0.37 (0.15–0.97)	0.043

Abbreviations: ERC = European Resuscitation Council; ICU = intensive care unit; mRS = modified Rankin Scale; OR = odds ratio.

a In patients with fewer than 2 unfavorable prognostic indicators according to ERC guidelines.

**Figure 2:**
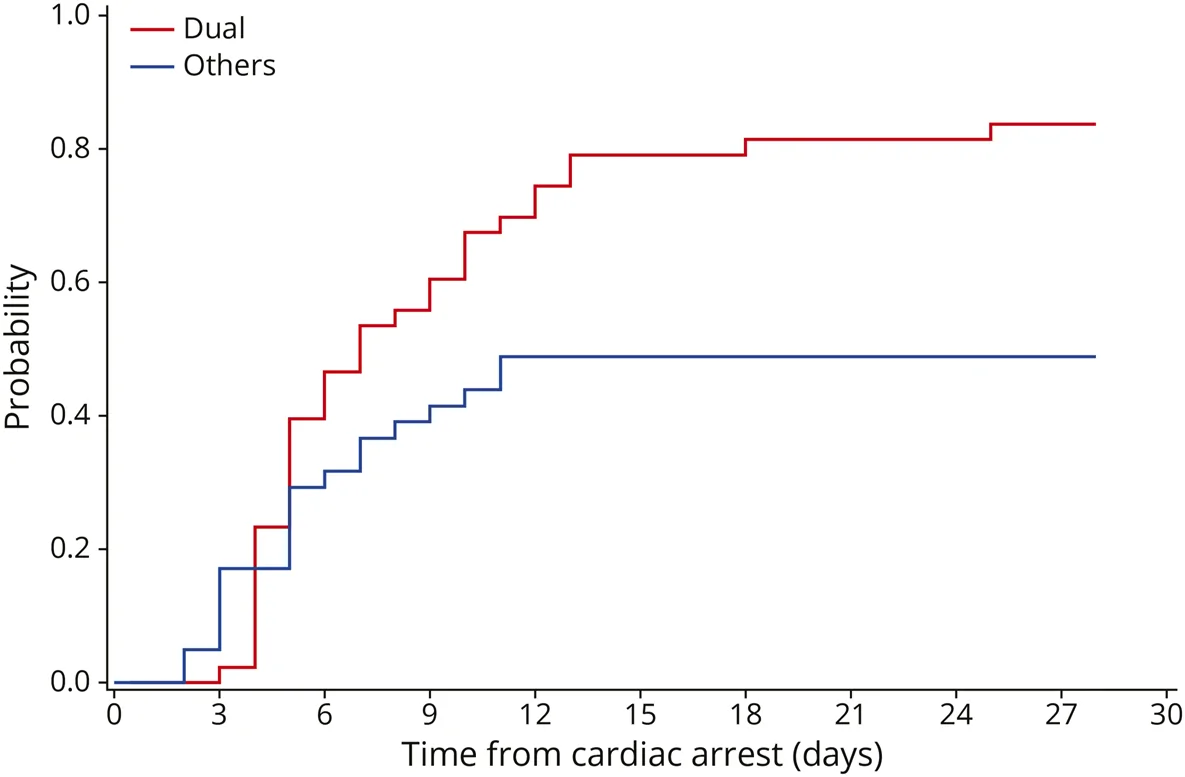
Cumulative Incidence of Status Epilepticus Resolution Cumulative incidence functions show the probability of resolution of super-refractory status epilepticus within 30 days after cardiac arrest in the DUAL and OTHERS groups, accounting for death as a competing event. Between-group differences were assessed using Gray test (*p* = 0.006).

### Secondary Outcomes

Among patients with fewer than 2 unfavorable prognostic indicators, early good neurologic outcome, defined as awakening to follow commands during the ICU stay, occurred in 18 of 32 patients (56.2%) in the DUAL group and 11 of 34 patients (32.3%) in the OTHERS group (OR 2.68; 95% CI 0.99–7.32; *p* = 0.053).

Three patients were lost to follow-up (DUAL, n = 1; OTHERS, n = 2). Long-term favorable neurologic outcome (mRS 0–3 at 6 months) was observed in 12 of 31 patients (38.7%) in the DUAL group and 8 of 32 patients (25.0%) in the OTHERS group (OR 1.89; 95% CI 0.64–5.56; *p* = 0.245). In the entire cohort, 6-month mortality was lower in the DUAL group (22/42, 52.4%) compared with the OTHERS group (29/39, 74.4%; OR 0.37; 95% CI 0.15–0.97; *p* = 0.043; Table 3). Time-to-event analyses for awakening and 6-month survival are presented in Figure 3, showing a higher cumulative incidence of awakening in favor of DUAL, as well as improved 6-month survival in the DUAL group (log-rank *p* = 0.003). Among patients who died during hospitalization, 19 (51%) died after withdrawal of life-sustaining therapy (WLST), 17 (46%) from progressive multiorgan failure, and 1 death was attributed to brain death.

**Figure 3:**
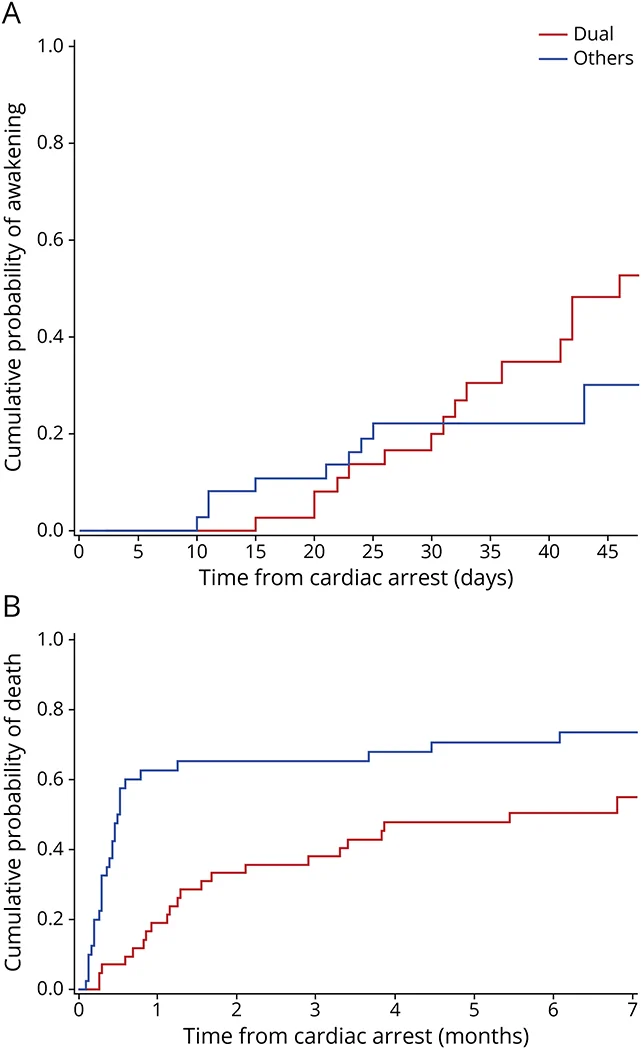
Early Neurologic Recovery and Long-Term Survival (A) Cumulative incidence of awakening to follow commands within 45 days. Difference in cumulative incidence +19.8% in favor of DUAL. (B) Cumulative incidence of death up to 6 months (log-rank *p* = 0.003).

### Safety Outcomes

Safety data are summarized in Table 4. Death during the ICU stay occurred in 12 patients (27.9%) in the DUAL group and 25 patients (60.9%) in the OTHERS group. GGT elevation greater than 3 times UNL was observed in 33 of 43 patients (76.7%) receiving DUAL therapy vs 11 of 41 (26.8%) in the OTHERS group. CK elevation (>10× UNL) occurred in 15 (34.8%) and 11 (32.3%) patients, respectively. One patient in the DUAL group experienced third-degree atrioventricular block; recurrence of cardiac arrest occurred in 2 patients (4.6%) in the DUAL group and 1 patient (2.4%) in the OTHERS group. No other adverse events potentially related to the neurologic treatment were observed.

**Table 4:** Safety Outcomes

	DUAL (n = 43)	OTHERS (n = 41)	OR (95% CI)	*p* Value
Death during the ICU stay, n (%)	12 (27.9)	25 (60.9)	0.24 (0.09–0.61)	0.003
GGT elevation (>3 times the UNL), n (%)	33 (76.7)	11 (26.8)	9.00 (3.34–24.19)	<0.001
Highest GGT value during the ICU stay, U/L, median (IQR)	624 (318–1,084)	557 (247–910)	NP	NP
CPK elevation (>10 times the UNL), n (%)	15 (34.8)	11 (32.3)	1.12 (0.43–2.90)	0.815
Third-degree atrioventricular block, n (%)	1 (2.3)	0 (0)	NP	NP
Recurrence of cardiac arrest, n (%)	2 (4.6)	1 (2.4)	NP	NP

Abbreviations: CPK = creatine kinase; GGT = gamma-glutamyl transferase; ICU = intensive care unit; NP = not performed; OR =odds ratio; UNL = upper normal limit.

### Single Antiglutamate Agents

The results from small cohorts of patients treated with ketamine alone (n = 6; without concomitant perampanel) and perampanel alone (n = 5; without concomitant ketamine) were also collected and are presented in eTable 3. Given the limited sample size, outcomes are reported descriptively; resolution of SRSE and GGT elevation appeared less frequent with either single antiglutamate drug than in the DUAL group.

## Discussion

In this multicenter cohort study, which included both retrospective and prospective phases, dual antiglutamate therapy with ketamine and perampanel was associated with a higher rate of resolution of SRSE compared with conventional treatments in selected postcardiac arrest patients, suggesting that this combination may represent a potential etiology-specific therapeutic strategy. The primary finding, 84% seizure resolution with dual antiglutamate therapy vs 49% with conventional combinations, represents a substantial and clinically relevant difference, supporting the rationale for future prospective randomized studies to confirm these results.

The superior efficacy of dual antiglutamate therapy likely reflects targeted intervention against the fundamental pathophysiology of postanoxic brain injury. Global cerebral ischemia triggers massive glutamate release, leading to excitotoxic cell death through both NMDA and AMPA receptor overactivation among other mechanisms.^8,22^ By simultaneously targeting NMDA and AMPA receptors, ketamine and perampanel may provide a complementary mechanism for interrupting this pathologic cascade, potentially offering advantages over conventional approaches targeting sodium channels or gamma-aminobutyric acid receptors.

Ketamine's neuroprotective properties extend beyond NMDA receptor antagonism, including anti-inflammatory effects and preservation of mitochondrial function.^23^ Perampanel, as the only selective noncompetitive AMPA receptor antagonist available for clinical use, specifically targets the receptor system responsible for the majority of fast excitatory neurotransmission in the brain.^24^

Notably, both drugs block their respective receptors in a noncompetitive manner, resulting in insurmountable inhibition of the glutamatergic system that is independent of glutamate levels released during the postanoxic event. Although both ketamine and perampanel have demonstrated efficacy when used individually in SE,^25,26^ their combined administration may enhance seizure control while providing additional neuroprotection during the vulnerable postresuscitation period.^27,28^

The timing and selection of patients for aggressive therapy remain critical. In our study, inclusion was restricted to patients meeting a minimum set of prognostic criteria, specifically preserved pupillary reflexes and bilateral N20 responses, while patients with GPDs were excluded. This careful selection likely contributed to the observed treatment success and aligns with the ERC guidelines advocating for multimodal assessment.^10^ For secondary outcomes focused on neurologic recovery, patients were further stratified according to ERC recommendations, excluding those with 2 or more unfavorable prognostic indicators, given their minimal likelihood of achieving a favorable outcome.

The 56% rate of neurologic awakening, defined as the ability to follow commands, in the dual-therapy group suggests a potential association between effective seizure control and clinically meaningful neurologic recovery. This observation is particularly relevant because early or late awakening during ICU stay has been associated with favorable long-term functional outcomes in postcardiac arrest patients.^29^ It should be noted, however, that long-term outcomes are influenced not only by neurologic recovery but also by systemic comorbidities, non-neurological causes of death, and cognitive or behavioral deficits that may be identified later, which may account for the apparent attenuation of neurologic benefit over time. Larger prospective studies are warranted to confirm the clinical advantages of dual antiglutamate therapy in postanoxic patients with SE refractory to first-line treatment.

These results extend beyond our previous study and preliminary single-center experience,^13,17,20^ providing multicenter confirmation of the feasibility of a dual antiglutamate approach. The 84% seizure resolution rate observed in this patient population exceeds that reported with conventional therapies in a recent large multicenter cohort,^14^ in which the resolution rate was 44%, closely corresponding to the 49% observed in the control arm of our study. Our findings are also consistent with a post hoc analysis of the TELSTAR trial,^11^ which showed a potential benefit of antiseizure treatment in patients with nonperiodic epileptiform patterns after cardiac arrest.

The safety profile of dual antiglutamate therapy, as outlined in the SUPER-CAT protocol, seems acceptable but deserves further investigation. The primary and frequent adverse event (>75% of cases) was biochemical evidence of cholestatic liver injury, which was reversible following perampanel tapering. This observation aligns with our prior preliminary report in 8 patients receiving perampanel oral loading, where hepatobiliary alterations were noted in 62.5% of cases.^17^ Although not immediately life threatening, this adverse event warrants further investigation in future clinical studies, with a careful risk-benefit assessment at the individual patient level, given the high burden of concomitant medications and the contribution of hypoxic liver injury in postcardiac arrest syndrome.

The major strength of our study lies in its multidisciplinary approach, with neurologists and intensivists working closely together, and in the application of an innovative treatment protocol in a patient population characterized by severe illness and high mortality. In addition, the multicenter design and the use of standardized definitions and prognostic approach aligned with the ACNS terminology and ERC guidelines enhance the potential generalizability of our findings. Collectively, this approach provides preliminary evidence of improved patient care, for both SE resolution and overall clinical outcomes. Several limitations should be acknowledged. First, the observational study design, including both retrospective and prospective phases, introduces potential selection bias, despite efforts to include consecutive patients meeting eligibility criteria and balanced baseline characteristics between study arms. Treatment assignment was not randomized; however, the absence of established evidence for optimal SRSE management likely minimized systematic treatment preferences. Second, although the sample size was adequate for the primary outcome, it may have limited power to detect differences in long-term clinical outcomes and rare adverse events. Third, the study was conducted at specialized centers with expertise in postcardiac arrest care and SE management, which may limit generalizability. The requirement for continuous EEG monitoring and meticulous multimodal prognostic assessment may not be universally available. Fourth, we adopted a conservative approach and decided to exclude patients with GPDs based on the results of the TELSTAR trial, which showed futility of treatment for GPDs after cardiac arrest. Therefore, the effect of DUAL on GPDs could not be assessed. Fifth, the potential influence of WLST decisions on neurologic outcomes and the risk of survivorship bias cannot be completely excluded. However, WLST decisions were made according to institutional protocols and contemporary neuroprognostication guidelines and were not initiated before 72 hours after cardiac arrest, minimizing the risk of premature treatment withdrawal. Sixth, this study specifically addresses postanoxic SE, which differs substantially from other etiologies of SRSE in terms of pathophysiology and prognosis; further studies are warranted to evaluate the efficacy and safety of the SUPER-CAT protocol in other patient populations. Seventh, perampanel plasma levels were not systematically measured. Given its long half-life (∼105 hours) in the absence of cytochrome P450 3A4 inducers, concentrations by day 5 may approximate those of a 6 mg/d regimen despite not reaching steady state. Future studies could consider higher loading doses, as reported in SE with suspected inflammatory etiology,^30^ and pharmacokinetics measurements.

In selected patients with SRSE after cardiac arrest, dual antiglutamate therapy with ketamine and perampanel was associated with higher rates of seizure resolution and showed a strong trend toward improved early neurologic recovery compared with conventional therapies, while its benefit on long-term outcomes remains uncertain. These findings support the rationale for a prospective, randomized controlled trial comparing dual antiglutamate therapy with the current standard of care in postanoxic SRSE, ideally conducted as a multinational effort.

## Glossary

ACNS: American Clinical Neurophysiology Society
ASM: antiseizure medication
CK: creatine kinase
eCRF: electronic case report form
ERC: European Resuscitation Council
GGT: gamma-glutamyl transferase
GPD: generalized periodic discharge
ICU: intensive care unit
IQR: interquartile range
mRS: modified Rankin Scale
NSE: neuron-specific enolase
OR: odds ratio
SE: status epilepticus
SRSE: super-refractory SE
SSEP: somatosensory evoked potential
SUPER-CAT: SUPER-refractory SE after Cardiac Arrest Treatment
TELSTAR: Treatment of ELectroencephalographic STatus Epilepticus After Cardiopulmonary Resuscitation
UNL: upper normal limit
WLST: withdrawal of life-sustaining therapy
